# Mortality in an ICU of a tertiary hospital

**DOI:** 10.1186/2197-425X-3-S1-A338

**Published:** 2015-10-01

**Authors:** AM Neto Real, A Araújo, I Coelho, N Lopes, T Lima Pereiro, M Abu-Hazim, L Pessoa, N Catorze

**Affiliations:** Internal Medicine Department, Abrantes, Portugal; ICU Department, CHMT, Abrantes, Portugal; ICU Department, Intensive Care Unit, CHMT, Abrantes, Portugal

## Introduction

Evaluation of mortality showed that in many ICU mortality in critically ill patients may range from 6.4% to 40% despite best care provided [1, 2]. This variability is considerable and persistent even after adjustment based on the characteristics of the patient on admission [3–8].

## Objectives and Methods

A retrospective observational study that aims to assess, analyze and characterize mortality in ICU -ABT, in 2014.

## Results

In this period, there were 608 admissions. The severity indices measured reached 48.5 points for SAPS II, and APACHE II 25.6 corresponding to a mortality rate of 43.8% and 56.9%, respectively. There were in total 170 deaths (27.9%). Of these, the majority were male (104 vs 66; 61% vs 39%) and the average age was 75.4 years and ranged between 37-97 years. The most prevalent age range was between 70-79 years (36%, n = 61). The average length of stay was 3.89 vs 2.99 days in patients who died. 54% (n = 92) of the deceased patients remained less than 24 hours in the ICU. Regarding the type of patient admitted, 82% presented a medical diagnosis (n = 139), 9.5% (n = 16) surgical-urgent and 8.5% (n = 15) surgical-elective diagnosis. The most prevalent primary diagnosis was septic shock which included 55 patients (32.4%) and cardio-respiratory arrest post-status in 13.5% (n = 23). The severity indices (APACHE II, and SAPS II) of the deceased patients reached 64 and 33 (75% and 78.6%) respectively.

## Conclusions

The data presented are consistent with the literature. Septic shock, most prevalent entity among the deceased, presented a mortality of up to 50%, so its strong representation is not surprising. It is to emphasize the fact that more than 50% of the deceased stayed less than 24 hours in the ICU, reflecting a late referral to the unit. The analysis of the severity index points to an estimated mortality higher than the observed, exposing a high quality of care provided.
